# Adaptation of POCT for pharmacies to reduce risk and optimize access to care in HIV, the APPROACH study protocol: examining acceptability and feasibility

**DOI:** 10.1186/s40814-018-0252-1

**Published:** 2018-02-27

**Authors:** Jason Kielly, Deborah V. Kelly, Christine Hughes, Kristine Day, Stephanie Hancock, Shabnam Asghari, Jacqueline Gahagan, Carlo Marra, Hai Nguyen

**Affiliations:** 1 School of Pharmacy, Memorial University of Newfoundland, 75 Tiffany Court, St. John’s, Newfoundland A1A 0L1 Canada; 2 Faculty of Pharmacy and Pharmaceutical Sciences, University of Alberta, Edmonton, Alberta Canada; 3 Faculty of Medicine, Memorial University, St. John’s, Newfoundland Canada; 4 School of Health and Human Performance, Dalhousie University, Halifax, Nova Scotia Canada; 5 School of Pharmacy, University of Otago, Dunedin, New Zealand

**Keywords:** HIV, Pharmacy, Pharmacist, Point of care test, Rapid HIV test, Implementation science

## Abstract

**Background:**

Approximately 1 in 5 Canadians with HIV are unaware of their status. In many provinces and especially rural communities, barriers to HIV testing include lack of access, privacy concerns, and stigma. The availability of HIV point-of-care testing (POCT) is limited across Canada. Pharmacists are well positioned to address barriers by offering rapid HIV POCT and facilitating linkage to care.

**Methods:**

We will use a type-2 hybrid implementation-effectiveness design to assess a pilot HIV POCT model in one urban and one rural pharmacy in each of two Canadian provinces over 6 months. In this feasibility trial the research aims include developing and assisting pharmacies in implementing the model, evaluating processes/determinants of program implementation, evaluating the model’s effects on client outcomes, preferences, and testing satisfaction. Using a community-based research approach, the research team will engage community stakeholders in each province including individuals with lived experience to inform the development of the pharmacy-based HIV testing model and support the research team throughout the study. A multipronged promotion campaign will be used to promote the study and facilitate recruitment. The pharmacy-based testing model will include pre/post-test counseling and linkage to care plans in addition to pharmacist-administered HIV POCT. Pharmacists will complete a comprehensive training program prior to implementing the testing model. Client demographics and satisfaction will be assessed by surveys and interviews. Pharmacists will document time required for testing and participate in a post-study focus group to discuss barriers/enablers. Implementation will be assessed qualitatively and quantitatively. The process of developing and implementing the model will be described using qualitative data and a logic model. Acceptability and barriers/enablers will be examined qualitatively based on survey responses. A preliminary costing assessment will consider the client, pharmacy, and government perspectives.

**Discussion:**

The results of this pilot will inform modifications to the HIV POCT model to optimize effectiveness and increase scalability. The study has national importance, providing valuable information on improving access to HIV testing. Future applications of this research may expand the role of pharmacists in offering POCT for other sexually transmitted/bloodborne infections as tests become available in Canada.

**Trial registration:**

Clinicaltrials.gov, NCT03210701

## Background

An estimated 21% of Canadians with human immunodeficiency virus (HIV) are unaware of their status [1]. Canadian guidelines recommend that HIV testing should be incorporated into routine medical care to normalize testing and achieve more timely diagnosis and linkages to care [2, 3]. However, practice has been slow to implement these recommendations, especially in smaller provinces where access to testing is limited [4]. Larger provinces in Canada, such as British Columbia, Ontario, and Quebec, have formalized provincial HIV testing programs and/or benefit from strong community-based networks to offer a variety of HIV testing options (e.g., community-based testing, peer testing, outreach workers). Other provinces have limited options for testing beyond traditional healthcare services (such as family physicians, emergency rooms, hospitals) and deal with barriers including cultural and geographic divides. Stigma and reluctance to discuss one’s sexual risk are significant barriers to requesting an HIV test, and these issues are particularly acute in smaller communities [5]. Low perceived risk of infection, inability/reluctance to access traditional healthcare services for testing, lack of testing outside of daytime business hours, delays for appointment times, and waiting for test results are also significant barriers [6, 7]. Those at highest risk, including people who inject drugs, men who have sex with men, and sex workers are especially affected by accessibility barriers [8]. Innovative approaches that make testing more accessible and overcome barriers may help to identify infections earlier, facilitate transition into care, reduce spread of infection, and reach the UNAIDS “90-90-90” target by 2020, to end the HIV epidemic [9, 10].

Point-of-care testing (POCT) for HIV has been shown to improve access to and uptake of HIV testing in areas where healthcare resources are limited [11, 12] and among high risk populations [11, 13, 14]. Two Canadian scoping reviews suggest the availability of HIV POCT has two significant public health impacts [11, 15]. First, POCT significantly improves the likelihood that patients will receive test results since results are conveniently available within minutes of testing. Second, it can help facilitate timely linkages to care as clients receiving a positive result are provided immediate post-test counseling and referrals to care. Evidence suggests HIV POCT is accepted by Canadians and is associated with high satisfaction rates [11, 15]. However the availability of HIV POCT is limited and variable across Canada––POCT is not available at all in the Atlantic Provinces (Newfoundland, Nova Scotia, New Brunswick and Prince Edward Island) or northern territories, and is limited outside of traditional healthcare settings in other provinces, especially in rural/remote areas. A national action plan has called for increased access to HIV POCT and research to determine whether HIV POCT matches the needs of the community [4].

### Rationale

Offering HIV POCT through community pharmacies may help improve access to testing and facilitate linkages to care, particularly in small towns lacking primary care clinics [11]. In rural areas, the pharmacist may be the only health provider. The scope of pharmacy practice has expanded rapidly and provision of POCT for a variety of conditions (e.g., influenza, group B strep) is becoming commonplace in many provinces. Increasingly, pharmacists offer a variety of individualized client-focused services, such as influenza vaccinations, in private counseling rooms. HIV POCT is easy to administer, and the results are available within minutes, making it ideal for use in the pharmacy setting. Pharmacists maintain close professional relationships with physicians and other health professionals in the community, which can support establishment of strong, effective linkage to care plans for those who receive a reactive POCT result or require testing for additional sexually transmitted or bloodborne infections (STBBI). These factors support offering HIV POCT in pharmacies as a means to improve access to and reduce the stigma associated with testing.

A pilot study in the USA demonstrated acceptability and feasibility of pharmacist-provided HIV POCT [16]. Notably, 42% of those tested indicated this was their first HIV test, and 66% had engaged in high-risk sexual activity in the preceding 6 months. The majority of participants (96%) and pharmacists (98.5%) reported favorable perceptions of the testing experience [16]. Enhanced testing programs that reach high-risk populations and the previously untested can play a key role in controlling the HIV epidemic by identifying those that are undiagnosed [3, 4].

While standard of care for HIV screening also includes testing for other STBBI due to their common modes of transmission, currently, there exists only one Health Canada-approved POCT for HIV (INSTI® HIV-1/HIV-2 rapid antibody test––bioLytical Laboratories Inc., Richmond, BC) and at the time of study design, there was no approved POCT for other STBBI in Canada.^1^ The logistics of offering STBBI screening in a pharmacy necessitates a POCT as venipuncture and genital swabs are impractical in this setting. Therefore, the scope of this study is limited to testing for HIV only. However, it will be valuable to demonstrate that POCT by pharmacists is an acceptable, feasible, and effective means to provide HIV testing, particularly in provinces where access is limited and in rural and non-urban areas where POCT has been less well studied. The results from this study may support establishment of an additional service delivery model to reach at risk individuals and serve as proof of concept for pharmacists to offer comprehensive STBBI testing as POCT for other STBBI become available in Canada.

Existing literature provides a strong foundation for conducting clinical effectiveness and implementation studies on HIV POCT in pharmacies and supports the design and evaluation of implementation strategies to help understand which tools and approaches work best [11, 15]. Consideration of contextual factors that may influence the effectiveness, adaptability, combination, and scalability of HIV POCT interventions in this novel setting will reduce the likelihood of a mismatch between the intervention and the demands of frontline pharmacy practice. Hybrid study designs are increasingly being used to address clinical effectiveness but utilize methods and procedures necessary to deliver and sustain interventions in real-world care settings. Hybrid designs provide more relaxed internal controls to improve generalizability [17]. Curran et al. advise not waiting for “perfect” effectiveness data before moving to implementation research, arguing that effectiveness data can be “backfilled” while implementation strategies are tested [18]. Rather than trying to control for real-world conditions or to remove their influence as causal effects, our approach is to use implementation science to understand and work within these real-world conditions.

The APPROACH study will use a type-2 hybrid study design [17] to help address the following important questions: *Can we identify characteristics of a pharmacy-based HIV POCT program to (a) reach people at increased risk of HIV, especially those who have never been tested; (b) be broadly adopted across different settings (including rural and urban); (c) be consistently implemented by different pharmacy staff members with moderate levels of training and expertise; (d) produce replicable and long-lasting effects (and minimal negative impacts); and (e) at a reasonable cost?*

### Study objectives

The main aim of the study is to assess the acceptability and feasibility of a multi-faceted, integrated model of HIV POCT in community pharmacies in both rural and urban settings in two Canadian provinces. The study has several objectives:

1. To assist community pharmacies in developing and implementing the model
  Partnering with pharmacists, staff, and managers to provide training and resource development, as well as identifying community resources to facilitate development of linkage to care plans individualized to each setting (region, province).
2. To evaluate processes and determinants of the HIV POCT program implementation in pharmacies to strengthen the intervention and its implementation. This will include:
  Assessing the acceptability of the intervention, barriers, and facilitators to implementation (number of HIV tests performed, workload/workflow issues, provider satisfaction, etc.)
  Understanding how implementation strategies and tools affect adoption, effectiveness, and fidelity and examining key determinants of sustainability.
3. To evaluate the effect of POCT implementation on client outcomes, preferences, and satisfaction with the testing experience.
4. To understand demographic, social, and behavioral characteristics of clients who seek HIV testing at pharmacies, including the proportion who are first-time testers, reasons for testing at a pharmacy (versus other testing options), and to assess whether these characteristics differ between those who access testing in urban versus rural pharmacies or by province.

A secondary aim will be to develop a framework to assess the costs of implementing POCT in pharmacies. Costs will include those at the client level, pharmacy level, and government level.

## Methods

### Study design

**Fig. 1:**
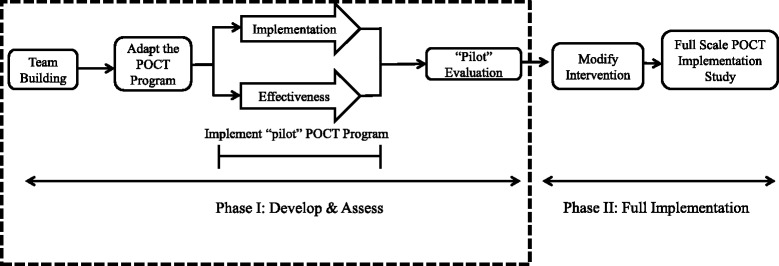
Study design

Using a type-2 hybrid design, we will develop and assess (phase I) the acceptability and feasibility of a multi-faceted, integrated, contextualized model of HIV POCT in one urban and one rural pharmacy in two Canadian provinces (Alberta (AB) and Newfoundland and Labrador (NL)) as part of a 6-month pilot study (see Fig. 1). HIV POCT has limited availability in AB and is currently unavailable in NL. This study design will be flexible, responsive, and capable of capturing changing elements at multiple points in time.

### Team development and governance

**Fig. 2:**
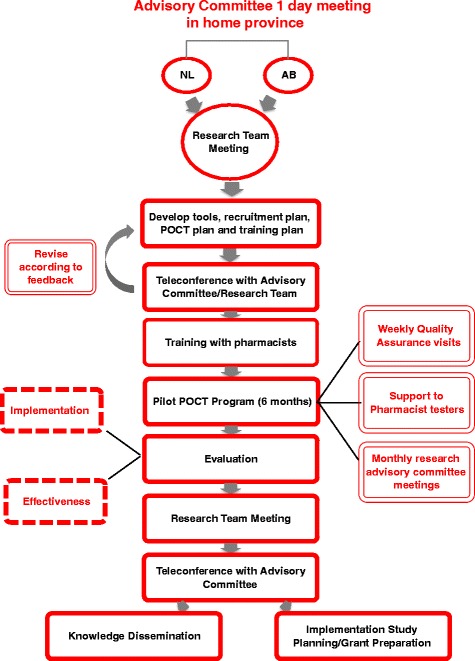
APPROACH study – Team Development and Study Governance

To ensure contextual factors are appropriately considered, the research team will be supported by provincial advisory committees (PAC). Using a community-based research approach, local stakeholders will be engaged and invited to form the PAC in each province. The PAC will be comprised of stakeholders including pharmacists and managers, HIV-experienced health workers, decision makers, and community representatives, including community-based organizations and individuals at risk or with lived experience. The PACs will provide advice and feedback to ensure the program developed is responsive to the needs of the local communities and will advise on issues and barriers to support implementation and uptake of POCT programs, the linkage to care plans for each of the four individual pharmacies, and the study promotion plan. Together, the research team and the PAC will function as one cohesive unit, shaping both the implementation and the effectiveness aims (see Fig. 2).

Members of the research team will hold meetings with PAC members in each province to identify contextual issues related to implementing a pharmacy-based POCT program, obtain advice on how to reach populations at risk (especially those who may not access testing through traditional testing sites), and obtain feedback on a proposed model for POCT testing by pharmacists. Following the two provincial PAC meetings, the research team will meet to review the findings from the PAC meetings, feedback on the proposed POCT model, recruitment plans, and linkage to care plan issues. Outputs from this meeting will include lists of tools, resources, and supports to be developed to support the POCT program for the pharmacy settings in each province, as well as training and supports required for pharmacists and staff offering the POCT. Consultation with the PAC will take place throughout the development process to inform revisions as necessary.

### POCT pilot program

#### Selection of pharmacy sites

Pharmacies in each province will be intentionally selected considering a variety of criteria including motivation to offer HIV testing as an expanded pharmacist service, private room available to provide testing, sufficient staffing to support the service, and ability to provide linkage to care (individualized based on each site) for patients who have a reactive HIV test result. One urban (population ≥ 100,000) and one rural (population < 100,000) pharmacy site from each province will be selected to participate in the study. Each pharmacy will be required to commit to designated testing hours each week that will be advertised on study promotional material, or to an appointment-based testing program if consistent, designated testing hours are not possible. Each participating pharmacy must have one pharmacist willing to undergo the complete training program and provide consent to participate in the study in order to offer testing and provide feedback on their experience at the end of the study.

#### Pharmacist training program

Participating pharmacists will complete an extensive training program consisting of four parts––an online self-study module, face-to-face training day, in-pharmacy competency assessment, and proficiency assessment. Prior to the training day, pharmacists will complete an online continuing education program which covers the basic elements of HIV POCT and watch an online video on the use of the INSTI® HIV-1/HIV-2 rapid antibody test (bioLytical Laboratories Inc.). The training day will consist of didactic and discussion sessions for the first half of the day covering the following topics: HIV 101, an overview of the study process and documentation tools; pre-/post-test counseling; and client supports. The second half of the day will be a hands-on session for pharmacists to learn how to use the INSTI® HIV-1/HIV-2 rapid antibody kits, interpret and explain results, quality control procedures, and practice consenting and counseling clients. The importance of client support, medical follow-up, and linkage to care will be a significant component of the training program.

Immediately prior to implementation of the POCT program, a pharmacy site visit will be conducted for competency assessment. The pharmacist will be observed completing the entire testing process and study procedure and their performance evaluated by a member of the research team using a checklist to ensure all steps are appropriately followed. Within 1 week, a proficiency assessment will be conducted in which each pharmacist tests a series of blinded samples provided by the provincial public health laboratories and completes a multiple choice test. The POCT and the multiple choice test results will be relayed to the public health laboratories to check accuracy.

#### Study population

The study population consists of adult clients in rural and urban areas of two Canadian provinces (AB and NL) who wish to be tested for HIV. Clients will not be specifically invited to participate in the study. Instead, a variety of promotional strategies will be used to promote the study to the general public and high-risk groups.

#### Study eligibility criteria

Adults who are 18 years of age who request HIV testing, who are not known to have HIV infection, and who provide their provincial healthcare number and informed consent will be eligible to participate in the study.

#### Promotional strategy

Clients will not be specifically approached or invited to participate in the study. Instead, a variety of techniques will be used to promote the study in each region and individuals will self-select and choose to approach the pharmacy to request HIV testing. Feedback from PAC members will inform development of specific promotional materials, such as posters, flyers, and post cards. Promotional materials will be posted and distributed to clients by community partners, including AIDS service organizations, organizations working with at-risk clients (such as people who inject drugs and sex workers), and through participating pharmacies. Posters will also be displayed in public gathering places in each community where testing is being offered, as well as neighboring communities.

Communications staff at each academic institution will develop and support a media relation campaign to promote the study (e.g., interviews, study website, media releases, social media promotion). Members of the research team will meet with community partners throughout the study to share interim findings (e.g., feedback from testers) and to maintain interest in the study to encourage ongoing promotion with each organization’s staff and clientele. A focused advertising campaign will be developed for online “hook up” sites to appeal to site users, encouraging them to get tested if they are having sex, and where testing is available through the study.

#### HIV POCT process––intervention

**Fig. 3:**
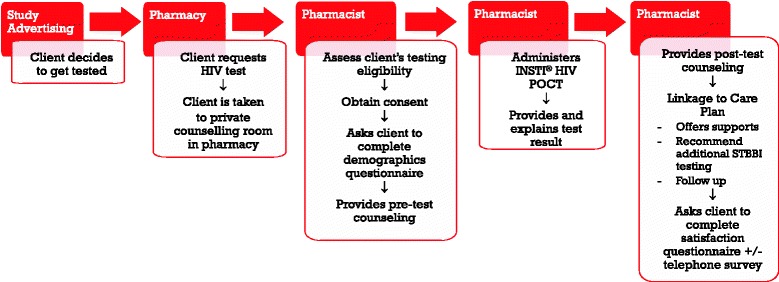
HIV POCT process in community pharmacies

Clients may request an HIV test at the pharmacy during advertised walk-in hours or by calling the pharmacy to arrange an appointment for testing. The client may request the test verbally or by passing a note at the pharmacy counter to enhance privacy. Pharmacy staff will bring the interested client to meet the pharmacist in a private counseling room. The pharmacist will then screen the client for eligibility, explain the study, and obtain informed written consent. Pre/post-test counseling and administration of the HIV test will be performed (see Fig. 3). The HIV test will be offered free of charge through the study.

**Fig. 4:**
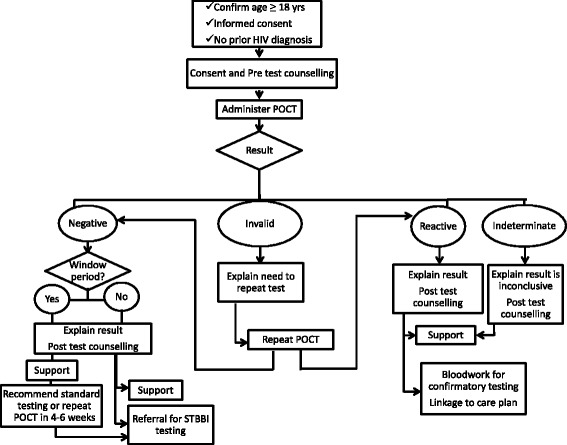
HIV POCT result protocol in community pharmacies

Pharmacists will administer the INSTI® HIV-1/HIV-2 rapid antibody test using a finger-prick blood sample. The test is easy to administer by trained personnel and has been used at numerous community-based testing sites across Canada by health professional and peer testers. The results are read within 60 s and are reported as reactive, non-reactive, indeterminate, or invalid (see Fig. 4). Reactive tests require confirmatory testing by the public health lab before a diagnosis of HIV infection is made. Clients with a reactive POCT result will receive immediate counseling, support and arrangements for confirmatory testing according to the pharmacy-specific linkage to care plan. The linkage to care plan also stipulates who will contact the client to communicate confirmatory test results, which is dependent on resources and the most appropriate health care provider in each community/province.

Linkage to care plans will be individualized for each pharmacy to utilize existing community resources to achieve the goal of quick, responsive, supportive linkage to care. Before leaving the pharmacy, clients with reactive POCT results will receive a bloodwork requisition from the pharmacist with counseling on where they can obtain confirmatory testing. Confirmatory bloodwork is ordered by the provincial HIV program nurse practitioner (NL) or medical director (AB) who will be notified by the pharmacist of the reactive POCT result. The pharmacist will provide the client’s healthcare number and contact information so that follow-up can occur with the client as soon as possible. In both provinces, local support services specific to each pharmacy will be identified, and pharmacists will undergo extensive training on how to provide client support, including helping the client identify their own supports and making a plan for the next 2–7 days while awaiting confirmatory test results.

For clients with non-reactive HIV POCT results, post-test counseling focuses on education regarding how to reduce their risk of HIV exposure in the future and recommending testing for additional STBBI. Clients will receive information on where they can access additional STBBI testing. Support services are also available to clients if needed. Participation in this trial or results of the HIV test will not be stored in the client’s pharmacy records or shared with the client’s family physician or third party payers.

#### Quality control

A process evaluation approach will be used to identify potential and actual influences on the conduct and quality of implementation as part of the fidelity assessment. Summative evaluations will assess patient-level health outcomes (effectiveness aims) and process/quality measures (implementation aims). Research staff will conduct biweekly site checks on each pharmacy (either in person, by telephone, and/or by Skype) to monitor uptake of testing services, ensure integrity of data collection, monitor fidelity to the study protocol, answer questions, and provide support to the pharmacists. The research team will also provide support to the pharmacists by being available for questions. A monthly conference call for all testing pharmacists with members of the research team will be offered to share experiences and challenges. Research team meetings will take place bimonthly, and liaison with PAC members will occur through individual meetings, calls, and teleconferences throughout the study.

#### Ethical and regulatory considerations

This pilot study was approved by the Newfoundland and Labrador Human Research Ethics Board (reference no. 2016.178) and the University of Alberta Health Research Ethics Board (reference no. Pro00066308). All participating pharmacists and clients included in the study sign consent forms as per ethical approval. The confidentiality of clients will be preserved as all participants will be allocated a unique identifier, and all trial data collected will be held in a linked anonymized form. Identifiable information will be stored separately from trial data.

#### Participant timeline

Patients will be recruited over a 6-month period in each of the two provinces, between February 2017 and October 2017. See Fig. 3 for an overview of the participant process.

## Data management and statistical analysis

### Data collection procedures and data to be collected

Participating pharmacists will complete de-identified data collection forms for each test performed to document the number of tests performed and time required for each step of the HIV testing process (e.g., informed consent, test setup and specimen collection, test processing, pre-, and post-test counseling, referral for linkage to care). At the end of the study, a focus group of pharmacists, pharmacy managers, and support staff (e.g., technicians and assistants) from each site will be held to determine their experiences and perceptions (attitudes toward offering HIV testing, perceptions of peer acceptability, opinions about readiness for implementation into practice, barriers, and enablers).

Qualitative and quantitative data will be collected. Clients will complete a de-identified survey prior to receiving their HIV test to collect demographic information (age, gender, ethnicity, marital status, education status, and income level), HIV risk factors, and testing history (first HIV test or not). After completion of the post-test counseling clients will complete a second brief survey to assess their perception of the testing experience (including factors that influenced their decision to get tested at the pharmacy and whether/where they would have sought HIV testing otherwise). The surveys will include questions to obtain data for the cost assessment. Client surveys will be collected in a sealed envelope, so the testing pharmacist cannot see the responses. Data will primarily be quantitative with some open-ended questions.

Clients will also be asked if they are willing to participate in a telephone interview within a week of their test to further explore qualitatively their perceptions about their testing experience. If they agree, the client will provide a phone number for a research assistant to call them at their preferred time to administer the interview. Data from this interview will be anonymous and not linked to their other data or test results.

### Sample size

As this was a pilot feasibility study, a sample size calculation was not performed. We estimate a minimum of 30 clients tested over the 6-month pilot to collect sufficient data to inform any modifications necessary prior to a broader scale implementation of the intervention in phase II of the study (Fig. 1). Data from key outcome measures will be used to determine the sample size for phase II.

### Data analysis plan

We will describe the process of developing and implementing the HIV POCT program in pharmacies in urban and rural settings in two provinces. This description will be developed from interviews, focus groups, notes from meetings and site visits, and other qualitative data collected during the study. Qualitative data (e.g., clients and providers: key informant interviews, focus groups, and open-ended surveys) will be transcribed verbatim. Two reviewers will independently review, code, thematically analyze, and subsequently compare their results. Agreement on final patterns and themes will be achieved in an iterative process, and discrepancies will be discussed and resolved. Qualitative data will be compared within and between settings and over time. Other sources of qualitative data (e.g., meeting minutes, site visit notes) will also be analyzed. We will develop a logic model to describe our program plan, the process, and summative evaluations to describe and evaluate implementation of the program.

To identify acceptability, barriers, and enablers, a variety of analytic approaches will be used. Setting measures (e.g., number of trained pharmacists, staff and managers, number of community resources identified, number of clients tested, number of POCT completed, successful linkage to care for clients with reactive results, workload/workflow issues, provider, and client satisfaction) will be described using frequency distributions and measures of central tendency. Performance measures (e.g., satisfaction, acceptability) from pharmacists, staff, and clients will be analyzed, using parametric and non-parametric tests. Comparisons will be made between urban and rural pharmacy test sites, within and between provinces.

To understand how the implementation strategies and tools affect adoption, fidelity, and effectiveness and to identify key determinants of sustainability, we will review the pharmacy settings before and after the implementation phase, and the pharmacy profile and logic model will be revisited and revised accordingly following implementation. We will conduct bivariate analyses to describe and test relationships between sustainability and characteristics of the settings and staff. Implementation fidelity will be assessed using the framework developed by Carroll et al. [19] with consideration of adherence, modifiers, and identification of essential components.

Descriptive analyses will be used to describe client outcomes, preferences, and satisfaction with the testing experience as well as demographic, social, and behavioral characteristics of clients who seek HIV testing at the pharmacies. Bivariate and multivariate analyses (appropriate for small sample size) will be conducted to assess whether the outcomes and client characteristics differ between those who access testing in urban versus rural pharmacies or between the two provinces. For each primary and secondary outcome, results and the estimated effect size and its precision (95% confidence interval) will be presented.

#### Cost assessment

Preliminary cost information will be collected from the patient surveys (e.g., distance traveled for testing, willingness to pay) and from the pharmacist (e.g., time required to complete testing). In addition, costs of implementation, delivery, and sustainability (including financial incentives) for pharmacies will be explored. These costs include the costs of training, consumables to perform the testing, administration, and reimbursement for the service (by the government or other third-party payers). Costs at the government level include procurement and distribution of the POC test kits, costs of confirmatory testing for those that tested as true and false positives using the POCT, and reimbursement (as described above). These costs will be critical in developing a framework for subsequent full economic evaluation of the POCT strategy versus the usual testing options available in each province.

### Protocol amendments

Any protocol amendments will be submitted to the NL Health Research Ethics Board and the University of Alberta Health Research Ethics Board for approval and noted in the registered protocol at ClinicalTrials.gov.

### Access to data

All investigators, research assistants, and data analysts will have access to the trial data.

## Trial status

Data collection is underway, but not yet complete. Data cleaning and analysis have not commenced.

## Discussion

In this study, we have used an effectiveness-implementation hybrid design that takes a dual focus a priori in assessing clinical effectiveness and implementation. The design does not replace large-scale trials that test effectiveness since it is a small scale under-powered study focusing on implementation of the intervention. Instead, it determines potential effectiveness for a main trial in assessing the feasibility of implementation.

The results of this implementation/effectiveness pilot study will inform modifications to the HIV POCT model for Canadian pharmacies to optimize effectiveness, increase scalability, and help construct a cost-effectiveness framework to assess sustainability. This innovative model of HIV testing will utilize existing resources (pharmacists) and infrastructure (e.g., public health, HIV programs) to improve access to testing for those who are not getting tested either because they cannot or choose not to access the traditional healthcare system. The impact of this study has provincial and national importance as it will provide valuable information about how to effectively and efficiently improve access to HIV testing in specific communities using a model that can be adapted for other communities and provinces within Canada, and beyond. If it is deemed to be feasible, effective, and acceptable, the pharmacy-based HIV testing model may become an important mechanism to increase testing among those at risk, facilitate timely diagnosis, and support client entry into care.

Future applications of this research may expand the role of pharmacists in offering POCT for other STBBI (e.g., hepatitis C, syphilis) as this testing technology becomes more widely available in Canada. Pharmacists could play a more direct role in HIV primary prevention by offering pre-exposure prophylaxis (PrEP) programs in provinces where pharmacists have authority to prescribe medications, provide education and counseling, offer regular HIV testing (by POCT), and order laboratory tests for confirmatory testing, monitor PrEP therapy, and provide timely linkages to care for those with reactive results and/or in need of additional STBBI testing. With the scope of pharmacist practice expanding rapidly in Canada as well as other countries, utilizing pharmacists to expand access to HIV testing may become an important adjunct to traditional/standard HIV testing options in many areas.

### Dissemination plans

There will be no publication restrictions for the full trial results, and publication will be sought in peer-reviewed journals. The authors plan to hold stakeholder meetings to disseminate study results, as well as present the results at local and national conferences.

## Abbreviations

HIV: Human immunodeficiency virus
PACs: Provincial advisory committees
POCT: Point-of-care testing
PrEP: Pre-exposure prophylaxis
STBBI: Sexually transmitted/bloodborne infection

## Footnotes

1 The OraQuick® HCV Rapid Antibody Test, a point-of-care test for hepatitis C (HCV) antibodies, was licensed by Health Canada in January 2017 for commercial sale in Canada.
